# MIO: microRNA target analysis system for immuno-oncology

**DOI:** 10.1093/bioinformatics/btac366

**Published:** 2022-06-01

**Authors:** Pablo Monfort-Lanzas, Raphael Gronauer, Leonie Madersbacher, Christoph Schatz, Dietmar Rieder, Hubert Hackl

**Affiliations:** Institute of Bioinformatics, Biocenter, Medical University of Innsbruck, 6020 Innsbruck, Austria; Institute of Medical Biochemistry, Biocenter, Medical University of Innsbruck, 6020 Innsbruck, Austria; Institute of Bioinformatics, Biocenter, Medical University of Innsbruck, 6020 Innsbruck, Austria; Institute of Bioinformatics, Biocenter, Medical University of Innsbruck, 6020 Innsbruck, Austria; Institute of Pathology, Neuropathology and Molecular Pathology, Medical University of Innsbruck, 6020 Innsbruck, Austria; Institute of Bioinformatics, Biocenter, Medical University of Innsbruck, 6020 Innsbruck, Austria; Institute of Bioinformatics, Biocenter, Medical University of Innsbruck, 6020 Innsbruck, Austria

## Abstract

**Summary:**

MicroRNAs have been shown to be able to modulate the tumor microenvironment and the immune response and hence could be interesting biomarkers and therapeutic targets in immuno-oncology; however, dedicated analysis tools are missing. Here, we present a user-friendly web platform MIO and a Python toolkit miopy integrating various methods for visualization and analysis of provided or custom bulk microRNA and gene expression data. We include regularized regression and survival analysis and provide information of 40 microRNA target prediction tools as well as a collection of curated immune related gene and microRNA signatures and processed TCGA data including estimations of infiltrated immune cells and the immunophenoscore. The integration of several machine learning methods enables the selection of prognostic and predictive microRNAs and gene interaction network biomarkers.

**Availability and implementation:**

https://mio.icbi.at, https://github.com/icbi-lab/mio and https://github.com/icbi-lab/miopy.

**Supplementary information:**

Supplementary data are available at *Bioinformatics* online.

## 1 Introduction

With the advent of cancer immunotherapies, the identification of modulators of the tumor immune microenvironment and the search for predictive biomarkers came into focus. There is growing evidence that cancer-derived microRNAs may play such a role and are involved in the cancer immune escape (Yi *et al.*, 2020). Over the last decade a variety of sequence-based target prediction tools and resources for experimental verified microRNA binding sites have been become available (Tokar *et al.*, 2018). The approaches were also extended to pan-cancer analyses (Dhawan *et al.*, 2018) and respective databases such as OncomiR (Wong *et al.*, 2018) or CancerMIRNome (Li *et al.*, 2021) have recently been developed. Although dedicated resources for cancer immunology exists (Charoentong *et al.*, 2017) comprehensive analyses tools and online resources for microRNA target analyses in this context are missing. For this purpose, we have developed the user-friendly versatile web platform MIO and the Python toolkit miopy.

## 2 MIO features and methods

MIO is a web application based on Python and the Django framework allowing a variety of microRNA target analyses of provided or custom datasets. MIO provides a set of preprocessed microRNAs and gene expression datasets as well as clinical meta data (including 33 cancer types from TCGA). A central component in MIO is the collection of published immune-related gene and microRNA signatures e.g. immune checkpoints and beyond the compendium of a variety of computational tools MIO provides a comprehensive searchable data/knowledge base for immuno-oncology data in the context of microRNA-gene targeting. For example, in cancer immunology and in the study of the tumor microenvironment, cell type-related marker genes or microRNAs as well as estimated immune cell infiltrates into the tumor and related microRNAs are of particular interest.

The integration of 40 target prediction tools (Supplementary Table S4) including information from experimentally validated databases allows robust identification of direct microRNA targets.

**Fig. 1.**
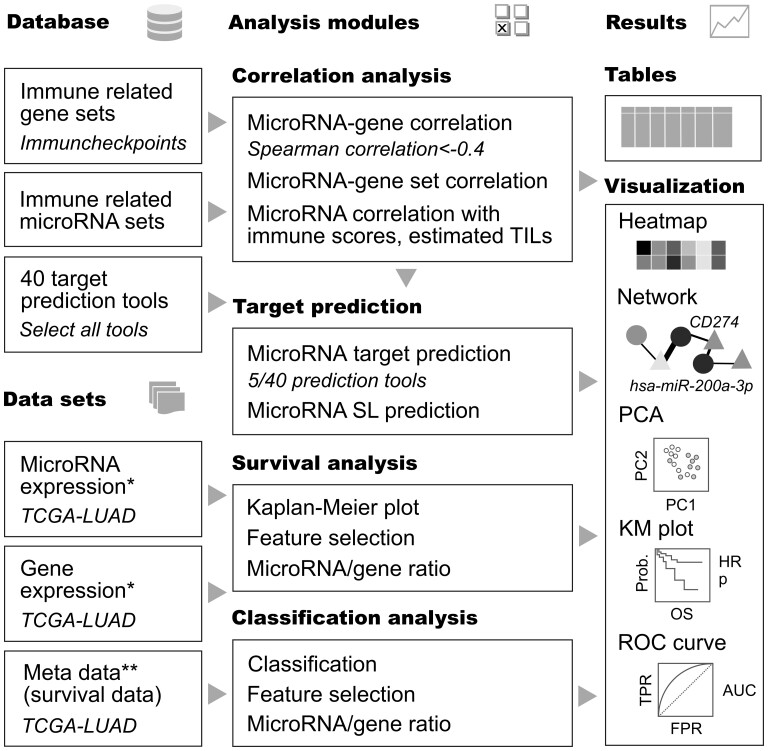
Schematic outline of the MIO components including database, datasets, analysis and filter modules and visualization of results. A workflow identifying microRNA target genes with correlation < −0.4 confirmed by 5 out of 40 target prediction tools within the immune checkpoints gene set in lung adenocarcinoma (TCGA-LUAD) is indicated (italic). *Public or custom gene and microRNA expression data provided in a matrix-based text file with log2 transformed normalized RNA sequencing data (e.g. using voom) or microarray data (e.g. using rma). For filtering differentially expressed genes or microRNAs RNA sequencing data needs to be either raw counts or voom transformed data. **Sample names (patient IDs) need to be matched with expression files

Four main types of analyses with respective filtering and visualization options are available (Fig. 1).

### 2.1 Correlation and regularized regression analysis

We use linear, nonlinear or rank-based correlation analysis methods as well as regularized regression models (Lasso, Ridge regression and Elastic Net) (Muniategui *et al.*, 2013) to compute the relationship between individual microRNAs and specific genes (e.g. from an immunity gene set), based on their normalized expression profile across all selected samples. The tool provides also a ranking (voting) method to integrate these analysis methods and helps to identify most promising candidates. In order to rule out random correlations, a background (null) model distribution of the correlation of a microRNA with 1000 randomly selected genes without evidence of any of the 40 target prediction tools is tested (List *et al.*, 2019). MicroRNA can be similarly associated with immune scores such as the immunophenoscore (IPS) (Charoentong *et al.*, 2017), or estimated immune cell infiltrations (Sturm *et al.*, 2019). The calculation of a score based on the number of supporting prediction tools for each patient (Buffa *et al.*, 2011) allows to identify and visualize the association of individual microRNAs regarding a complete pathway or gene set.

### 2.2 MicroRNA target prediction

A combination of 40 target prediction tools including five tools with experimental evidence can be used to identify targets for specified microRNAs or filtered from correlation results and visualized as an interactive heat map as well as a microRNA-gene network indicating the (opposing) effects of a microRNA and its target gene on survival.

MIO provides also the possibility to predict synthetic lethal microRNA target genes based on a pre-calculated list of synthetic lethal gene pairs (Lee *et al.*, 2018), to identify all (off-)targets of a microRNA and to test for over-representation.

### 2.3 Identification of prognostic biomarkers and survival analyses

MIO allows to identify prognostic biomarker signatures and ranking of features (microRNA, genes or microRNA/gene ratios for specified immune signatures) based on presence in the top candidates during a *k*-fold cross-validation procedure using different machine learning approaches (gradient boosting, support vector machines, penalized Cox regression). A Kaplan–Meier survival analysis can be performed by dividing patient groups into high versus low microRNA or gene expression by a selected percentile such as median or optimized dichotomization.

### 2.4 Identification of predictive biomarkers and classification

In order to identify predictive (network) biomarkers between two levels of a selected clinical parameter (e.g. response versus non-response) the feature selection process is very similar as for identifying prognostic features using a number of algorithms and ensemble methods such as adaptive boosting (Lopez-Rincon *et al.*, 2019). Using a set of selected features and a particular model (random forest, logistic regression or support vector machine) classification can be performed and information about the performance across *k*-fold cross-validation, ROC curve and principal component analysis with selected features are provided.

## 3 Results and use cases

A specific use case is the identification of microRNAs targeting immune modulators or checkpoints. As most information is available for microRNAs targeting PD-L1 (CD274), we compared these results with those generated using MIO for different cancer entities (Use Case S1). In this context, we identified the hsa-miR-150-5p/CD276 ratio as one of the most prognostic in lung adenocarcinoma (TCGA-LUAD), whereby hsa-miR-150-5p is targeting CD276 (also known as B7-H3) and is inversely associated with overall survival (Use Case S2). Deficient or down-regulated genes of the antigen processing and presentation machinery have been shown as predictive marker for response to cancer immunotherapy. Therefore, we studied how these genes are affected by microRNAs (Use Case S3). In order to identify a microRNA signature predictive of the microsatellite instability subtype in colorectal cancer, we performed feature selection and classification analyses in the TCGA cohort and validated the results in the CPTAC-2 cohort (Use Case S4). In Use Case S5, we could identify microRNAs potentially targeting genes, which are synthetic lethal to immune (therapy) essential genes indicating cancer therapeutic vulnerabilities. Finally, in Use Case S6, we could associate microRNAs—e.g. hsa-miR-223-3p—with the IPS and estimated infiltrated immune cells in the ovarian cancer cohort (TCGA-OV).

## 4 Conclusion

There are some limitations as several approaches that are also relevant to immuno-oncology applications are not currently implemented, for instance, a full microRNA-gene network (including transcription factors) (Nersisyan *et al.*, 2021) or analysis of individual target sites that allow investigation of microRNA cooperativity (Lai *et al.*, 2019), but these examples will be considered as analysis modules in future versions. Ultimately, the integrated analyses and machine learning methods available in MIO together with the data provided will help cancer researchers and immuno-oncologists to identify microRNAs and their target genes as potential biomarkers or candidates for cancer immunotherapy.

## Funding

This research was funded in whole, or in part, by the Austrian Science Fund (FWF) [P34783], the National Bank of Austria (OeNB) [18279 to H.H.], and the ERASMUS+ program from the University of Valencia to P.M.

*Conflict of Interest*: none declared.

## Supplementary Material

btac366_Supplementary_Material
