# Supplementary material for: MIO: microRNA target analysis system for immuno-oncology
Source: Bioinformatics. 2022 Jun 1;38(14):3665–7. doi: 10.1093/bioinformatics/btac366 (PMC9272810; doi:10.1093/bioinformatics/btac366)
Supplement: btac366_Supplementary_Material [file btac366_supplementary_material.pdf]

Supplementary Information

MIO: MicroRNA target analysis system for Immuno-Oncology.

Pablo Monfort-Lanzas[1,2], Raphael Gronauer[1], Leonie Madersbacher[1], Christoph Schatz[3], Dietmar Rieder[1], and Hubert Hackl[1*]

[1]Institute of Bioinformatics, Biocenter, Medical University of Innsbruck, Innrain 80, 6020 Innsbruck, Austria, [2]Institute of Medical Biochemistry, Biocenter, Medical University of Innsbruck, Innrain 80, 6020 Innsbruck, Austria, [3]Institute of Pathology, Neuropathology and Molecular Pathology, Medical University of Innsbruck, Müllerstraße 44, 6020 Innsbruck, Austria

*To whom correspondence should be addressed.

METHODS

1 Implementation

MIO is a Python (version 3.8.8) and R (version 4.1.0) application developed with the Django (version 3.2) framework. Since microRNA/Gene correlation or the feature selection is computational intensive and highly time consuming, MIO uses queueing through Django_rq (version 2.4.1) to handle the whole analysis in Django. This allows the user to still use MIO while the analysis is processing, and at the same time avoid overloading the system. When the results are ready, the analysis is visible on the main page of the user session. The backend consists of different scripts developed in Python using the scikit-learn (version 0.24.2) library, which is flexible and easy to parallelize. In order to handle the differential expression analysis and to normalize the expression file a customized R script is used. The object-relational database system PostgreSQL (version 14.1) is used to manage the user information, the results, workflows, queues, gene sets, microRNA sets, and the gene and microRNA annotations. Gene annotations were retrieved from the NCBI Gene database (gene_info.gz, Dec 2020), and microRNA annotation from miRBase Release 22.1 (Kozomara *et al.*, 2019). MIO uses Nginx to act as a reverse proxy for Gunicorn to handle client requests as well as serve up static files. The graphical user interface of MIO is based on Bootstrap 4. All the plots used in MIO are generated using Plotly (version 4.14.3) and rendered as HTML code. Interaction networks were built with networkx (version 2.5.1) in Python and visualized using Cytoscape.js. Finally, the MIO environment and code were packaged in a Docker image (version 5.0.0), and all elements were integrated using Docker Compose (version 1.29.2).

2 Availability and Usermanagement

MIO can be easily accessed online at http://mio.icbi.at without any login procedure and all provided public analyses can be viewed. The user can also sign in as an (automatically generated) anonymized user without the need of providing any personal information or as registered user providing only *Name* and *Email address* allowing to analyze custom data and all provided processed data without any restrictions. Data from anonymized user will be automatically removed from the server after seven days. In MIO, under the Help menu there is an example dataset available and also the manual can be viewed or downloaded (http://mio.icbi.at/staticfiles/media/MIO_Manual.pdf). The code of the MIO software is available under MIT license at the github repository https://github.com/icbi-lab/mio.

The python library miopy includes the same functionality and data sets as the web-based system MIO. The library miopy together with its documentation/Jupyter notebook and source code is available at https://github.com/icbi-lab/miopy.

3 Datasets

In MIO and miopy it is possible to analyze custom data consisting of one file with gene expression data, one file with microRNA expression data. An optional metafile with clinical data, including for instance survival data in months and the corresponding event data, may also be provided. Patient sample identifiers need to be identical. The analyses are not limited to specific applications, however, only human data is supported. In order to get the most benefit for immuno-oncology we have integrated a number of publicly available datasets, including immune related gene and microRNA sets as well as clinical meta data, gene and microRNA expression from large patient cohorts of various cancer types e.g. from The Cancer Genome Atlas (TCGA). This data collection is available for all users and will be expanded regularly. In order to get fast access to the results different analyses from these data are pre-calculated.

Public gene sets

We have collected a published/curated set of 231 immune related gene signatures (Table S1). They include signatures specifically related to immune cell types frequently present in the tumor microenvironment, immune related signatures such as antigen processing and presentation, immune modulators and immune checkpoints, chemokines and cytokines and their receptors. Since immunotherapy studies include few patients there are no consistent biomarkers for checkpoint blocker (ICB) therapy available, although recent meta-analyses (Litchfield *et al.*, 2021) indicate clonal mutational burden as most significant predictor, CD8, PD-L1(CD274), and T cell inflamed gene expression signature is potentially predictive as well. Hence, we include also various signatures with possible predictive value for immunotherapy response.

Table S1: Resources of public immune related gene sets

SETS	DESCRIPTION	TOOL/DATABASE	REFERENCE
24	Intratumoral immune cells (CRC)		(Bindea *et al.*, 2013)
10	Immune cell types	quanTIseq	(Finotello *et al.*, 2019),
28	Pan cancer immune metagenes	TCIA	(Charoentong *et al.*, 2017)
1	Cancer germline antigens	TCIA	(Charoentong *et al.*, 2017)
6	Antigen processing, immune modulators	TCIA	(Charoentong *et al.*, 2017)
1	Immunophenoscore (IPS)	TCIA	(Charoentong *et al.*, 2017)
10	Immune cell marker genes	MCPcounter	(Becht *et al.*, 2016)
31	TME gene expression signatures	Athena	(Bagaev *et al.*, 2021)
15	Immune, Chemokine, Cytokine, APM	ImmPort	(S. Bhattacharya *et al.*, 2014)
10	Immune signatures	ImSig	(Nirmal *et al.*, 2018)
2	ICB prediction (Cox interaction with CTL)	TIDE	(Jiang *et al.*, 2018)
3	CTL, CD8, IFNG	TIDE	(Jiang *et al.*, 2018)
1	Relations between ICP gene pairs	IMPRES	(Auslander *et al.*, 2018)
2	Intratumoral stimulatory DC, NK		(Barry *et al.*, 2018)
3	IFNG, T cell inflamed		(Ayers *et al.*, 2017)
1	IFNG		(Higgs *et al.*, 2018)
1	T cell inflamed		(Spranger *et al.*, 2016)
1	T cell exclusion signature		(Jerby-Arnon *et al.*, 2018)
1	Tumor inflamation signature (TIS)		(Danaher *et al.*, 2018)
1	PD-L1 synthetic pathway		(Danbaran *et al.*, 2020)

1	Tertiary lymphoid structures (TLS)		(Cabrita *et al.*, 2020)
1	Immune signatures (IS), aCTLA4		(Ock *et al.*, 2017)
1	Cytolytic activity		(Rooney *et al.*, 2015)
50	Hallmark collection	MSigDB	(Liberzon *et al.*, 2015)
1	Immune essential genes		(Patel *et al.*, 2017)
20	DNA damage and repair		(Knijnenburg *et al.*, 2018)
5	Curated immune sets		ICBI/CBIO

Public microRNA sets

In order to focus on specific sets of microRNAs we have included published information on microRNAs, which potentially target immune checkpoints/immune modulators, are related to immune cell types in the tumor microenvironment, or are involved in tumor immune escape (Table S2).

Table S2: Resources of public immune related microRNA sets

NR MIRNA	DESCRIPTION	REFERENCE
28	Lymphocyte microRNA Signature	(Incorvaia *et al.*, 2020)
9	ImmiRSig	(Xu *et al.*, 2021),
54	Tumor immune escape	(Eichmüller *et al.*, 2017)
31	Immunomodulatory microRNAs up in cancer	(Omar *et al.*, 2019)
23	Immunomodulatory microRNAs down in cancer	(Omar *et al.*, 2019)
14	Tumor associated miRNAs affecting tumor immune escape	(Hirschberger *et al.*, 2018)
8	Immuno-miRs	(Hirschberger *et al.*, 2018)
11	Immune checkpoint related microRNAs	(Smolle *et al.*, 2017)
49	Immune checkpoints	(Yang *et al.*, 2018)
36	Immune regulation	(Cortez *et al.*, 2019)
5	Regulating antigen processing and presentation	(Yi *et al.*, 2020)
28	Regulating NKG2D ligands	(Yi *et al.*, 2020)
25	Regulating PD-L1	(Yi *et al.*, 2020)
21	Regulating immune evasion via exosomes	(Yi *et al.*, 2020)
16	Tumor-immune communication	(Cho *et al.*, 2020)
35	MiRNA related with TAM in tumor progression	(Xing *et al.*, 2021)
23	MiRNA related with NK cells in tumor progression	(Xing *et al.*, 2021)
15	MiRNA related with MDSC in tumor progression	(Xing *et al.*, 2021)
9	MiRNA influences the tumor process by modulating Treg	(Xing *et al.*, 2021)
6	MiRNA roles in granulocytes	(Xing *et al.*, 2021)
10	MiRNA Roles in DC	(Xing *et al.*, 2021)
8	MiRNAs mediate platinum resistance in OC	(Zou *et al.*, 2021)
19	MiRNA mediated immune escape in OC	(Zou *et al.*, 2021)
46	Immunoregulators	(Lone *et al.*, 2021)
20	MicroRNA targeting PD-L1	(Danbaran *et al.*, 2020)
207	MicroRNA targeting PD-L1 synthesis pathway	(Danbaran *et al.*, 2020)

Expression datasets with meta information

We have downloaded pre-processed gene expression and microRNA expression data (RNAseq raw counts) as well as clinical metadata from 33 cancer types from The Cancer Genome Atlas (TCGA) datasets (Table S3) via the Genomic Data Commons Data Portal (https://portal.gdc.cancer.gov) using the *GDCRNATools* R package (Li *et al.*, 2018). Gene IDs were updated to official gene symbols (HGNC) and data were normalized by voom transformation (Law *et al.*, 2014). Expression data from the Gene Expression Omnibus (GEO) were downloaded using the R package GEOquery. Microarray data were normalized using the

rma from the R package *affy* and probe sets with highest mean expression levels were kept and annotated according official gene symbols (HGNC) and mature miRBase IDs. For ovarian cancer, another data set from an Australian patient cohort were downloaded from the International Cancer Genome Consortium (ICGC) (https://dcc.icgc.org) and processed accordingly.

Table S3: List of included cancer datasets with paired gene expression and miRNA expression data

N	DB	ID	DESCRIPTION	REFERENCE
322	TCGA	OV	Ovarian serous cystadenocarcinoma	(CGARN *et al.*, 2011)
93	ICGC	OV-AU	Ovarian cancer	https://dcc.icgc.org/
79	TCGA	ACC	Adrenocortical carcinoma	(Zheng *et al.*, 2016)
408	TCGA	BLCA	Bladder urothelial carcinoma	(Robertson, Kim, *et al.*, 2017)
1093	TCGA	BRCA	Breast invasive carcinoma	(CGARN *et al*., 2012)
304	TCGA	CESC	Cervical squamous cell carcinoma	(CGARN *et al*., 2017)
36	TCGA	CHOL	Cholangiocarcinoma	(Farshidfar *et al.*, 2017)
285	TCGA	COAD	Colon adenocarcinoma	(CGARN *et al*., 2012a)
48	TCGA	DLBC	Diffuse large B-cell lymphoma	(Schmitz *et al.*, 2018)
184	TCGA	ESCA	Esophageal carcinoma	(CGARN *et al*., 2017a)
153	TCGA	GBM	Glioblastoma multiforme	(CGARN *et al*., 2008)
520	TCGA	HNSC	Head and neck squamous cell carcinoma	(CGARN *et al*., 2015)
66	TCGA	KICH	Kidney chromophobe	(Davis *et al.*, 2014)
533	TCGA	KIRC	Kidney renal clear cell carcinoma	(CGARN *et al*., 2013)
290	TCGA	KIRP	Kidney renal papillary cell carcinoma	(CGARN, 2016)
151	TCGA	LAML	Acute myeolid leukemia	(CGARN *et al.*, 2013)
516	TCGA	LGG	Brain lower grade glioma	(CGARN, 2015b)
371	TCGA	LIHC	Liver hepatocellular carcinoma	(CGARN *et al*, 2017b)
515	TCGA	LUAD	Lung adenocarcinoma	(CGARN *et al.*, 2014)
501	TCGA	LUSC	Lung squamous cell carcinoma	(CGARN, 2012a)
87	TCGA	MESO	Mesothelioma	(Hmeljak *et al.*, 2018)
178	TCGA	PAAD	Pancreatic adenocarcinoma	(CGARN *et al*., 2017c)
179	TCGA	PCPG	Pheochromocytoma and paraganglioma	(Fishbein *et al.*, 2017)
497	TCGA	PRAD	Prostate adenocarcinoma	(CGARN *et al*., 2015b)
94	TCGA	READ	Rectum adenocarcinoma	(CGARN, 2012b)
259	TCGA	SARC	Sarcoma	(CGARN, 2017a)
103	TCGA	SKCM	Skin cutaneous melanoma	(Akbani *et al.*, 2015)
415	TCGA	STAD	Stomach adenocarcinoma	(CGARN, 2014a)
150	TCGA	TGCT	Testicular germ cell tumors	(Shen *et al.*, 2018)
501	TCGA	THCA	Thyroid carcinoma	(CGARN, 2014c)
120	TCGA	THYM	Thymoma	(Radovich *et al.*, 2018)
176	TCGA	UCEC	Uterine corpus endometrial carcinoma	(CGARN, 2013b)
57	TCGA	UCS	Uterine carcinosarcoma	(Cherniack *et al.*, 2017)
80	TCGA	UVM	Uveal Melanoma	(Robertson, Shih, *et al.*, 2017)
65	GEO	GSE29623	Colorectal cancer	(Chen *et al.*, 2012)
17	GEO	GSE35602	Colorectal cancer (tumor and stroma)	(Nishida *et al.*, 2012)
105	TCGA	CPTAC-2	Colon cancer	(Vasaikar et al., 2019)

CGARN, Cancer Genome Atlas Research Network; GEO, Gene Expression Omnibus, TCGA, The Cancer Genome Atlas; ICGC, International Cancer Genome Consortium; DB, database; N, number of patients; ID, cancer type or database identifier;

These public datasets (available for all users) can be used for all type of analyses and filtered based on specific levels of a selected variable, which is provided as a column in the clinical meta data file. For instance, cancer datasets can be filtered for primary tumor samples or for specific tumor stages. There is also the possibility to perform differential expression analyses between two specified levels using *limma* (Ritchie *et al.*, 2015). For example, only significantly

differential expressed genes and microRNAs which are more than two-fold changed between paired samples from normal tissue and primary tumor tissue - at a false discovery rate (Benjamini-Hochberg adjusted p-values) <0.1 - can be selected for further analyses.

4 Integration of microRNA target prediction tools

A variety of target prediction tools with different advantages using sequenced based computational methods and experimental verified targets have been developed over the years and we integrated established tools in order to enable the identification of direct microRNA target genes (Table S4). We downloaded and preprocessed the data according to the information summarized in Table S5. We were taking advantage of a recent approach, MirDIP, which includes a collection of 30 resources (Tokar *et al.*, 2018) and the R/Bioconductor package *multiMIR* (Ru *et al.*, 2014). All gene and transcript information were mapped to official gene symbols (HGNC) and all microRNA names/identifiers were mapped to mature microRNA ID from miRBase Release 22.1 (Kozomara *et al.*, 2019). Data were transformed into a matrix with binary encoding the interaction between genes (rows) and microRNAs according to the respective prediction tool (columns). We also analyzed individual microRNA targets with the experimentally validated microRNA target database DIANA Tarbase v8 (Karagkouni *et al.*, 2018) via the online service (http://carolina.imis.athena-innovation.gr). A logic combination (and/or) of these 40 microRNA target prediction databases or the number of supporting databases can be applied to specified candidates or to filtered candidates from previously performed correlation analyses.

Table S4: List of target prediction tools

TOOL	URL	REFERENCE
BCmicrO	http://compgenomics.utsa.edu/gene/gene_1.php	(Yue *et al.*, 2012)
CoMeTa	https://cometa.tigem.it/site/index.php	(Gennarino *et al.*, 2012)
Cupid	http://cupidtool.sourceforge.net	(Chiu *et al.*, 2015)
DIANA-microT	http://diana.imis.athena-innovation.gr/DianaTools	(Paraskevopoulou *et al.*, 2013)
ElMMo3	http://www.clipz.unibas.ch//ElMMo3	(Gaidatzis *et al.*, 2007)
GenMir++	http://www.psi.toronto.edu/genmir	(Huang *et al.*, 2007)
MAMI	https://mami.med.harvard.edu/	---
MBStar	https://www.isical.ac.in/~bioinfo_miu/MBStar	(Bandyopadhyay *et al.*, 2015)
MicroCosm	http://www.ebi.ac.uk/enright-srv/microcosm/cgi-bin/targets/v5	(Griffiths-Jones *et al.*, 2008)
miR2Disease	http://www.mir2disease.org	(Jiang *et al.*, 2009)
MirAncesTar	https://www.cs.mcgill.ca/~blanchem/mirancestar	(Leclercq *et al.*, 2017)
mirBase	http://www.mirbase.org	(Kozomara and Griffiths-Jones, 2014)
miRanda	http://www.mircrorna.org	(John *et al.*, 2004)
miRBridge	http://mirsystem.cgm.ntu.edu.tw	(Tsang *et al.*, 2010)
miRcode	http://www.mircode.org	(Jeggari *et al.*, 2012)
mirCoX	http://wrenlab.org/mirCoX	(Giles *et al.*, 2013)
miRDsnp	http://mirdsnp.ccr.buffalo.edu/download.php	(Bruno *et al.*, 2012)
miRDB	http://mirdb.org/download.html	(Chen and Wang, 2020)
mirDIP	http://ophid.utoronto.ca/mirDIP	(Tokar *et al.*, 2018)
miRecords†	http://c1.accurascience.com/miRecords/download.php	(Xiao *et al.*, 2009)
MirGate	http://mirgate.bioinfo.cnio.es	(Andrés-León *et al.*, 2015)
miRMap	https://mirmap.ezlab.org/downloads/mirmap201301e	(Vejnar and Zdobnov, 2012)
MirSNP	http://bioinfo.bjmu.edu.cn/mirsnp/search	(Liu *et al.*, 2012)
MirTar	http://mirtar.mbc.nctu.edu.tw	(Hsu *et al.*, 2011)
miRTar2GO	http://www.mirtar2go.org	(Ahadi *et al.*, 2017)
miRTarBase†	https://mirtarbase.cuhk.edu.cn/~miRTarBase/miRTarBase_2019	(Huang *et al.*, 2020)
mirWalk3	http://mirwalk.umm.uni-heidelberg.de/resources	(Sticht *et al.*, 2018)
Mirza-G	http://www.clipz.unibas.ch/index.php?r=tools/sub/mirza_g	(Gumienny and Zavolan, 2015)

MultiMiTar	https://www.isical.ac.in/~bioinfo_miu/multimitar-download.htm	(Mitra and Bandyopadhyay, 2011)
OncomiRDB	http://lifeome.net/database/oncomirdb/	(Wang et al., 2014)
PACCMIT	https://www.epfl.ch/labs/lcpt/research	(Marín *et al.*, 2012)
PharmacoMir	http://www.pharmaco-mir.org	(Rukov *et al.*, 2014)
PITA	https://genie.weizmann.ac.il/pubs/mir07/mir07_data.html	(Kertesz *et al.*, 2007)
PolymiRTS	https://compbio.uthsc.edu/miRSNP/download/PolymiRTS3.0	(A. Bhattacharya *et al.*, 2014)
RNA22	https://cm.jefferson.edu/rna22-full-sets-of-predictions	(Loher and Rigoutsos, 2012)
RPmirDP	https://doi.org/10.5683/SP2/LD8JKJ	(Kyrollos *et al.*, 2020)
StarBase†	http://starbase.sysu.edu.cn	(Li *et al.*, 2014)
TargetRank	http://hollywood.mit.edu/targetrank/downloads.html	(Nielsen *et al.*, 2007)
TargetScan	http://www.targetscan.org	(Agarwal *et al.*, 2015)
TargetSpy	http://webclu.bio.wzw.tum.de/targetspy/index.php?down=true	(Sturm *et al.*, 2010)

† databases including validated microRNA targets

Table S5: Resources, download and preprocessing of microRNA target prediction tools

TOOL	RESOURCE/DOWNLOAD	COMMENTS
BCmicrO	MIRDIP_MICRORNA_E-Version4.0.txt	
CoMeTa	MIRDIP_MICRORNA_E-Version4.0.txt	
Cupid	MIRDIP_MICRORNA_E-Version4.0.txt	
DIANAmicroT	http://diana.imis.athena-innovation.gr/DianaTools	v5, miTG score >= 0.7
ElMMo3	MIRDIP_MICRORNA_E-Version4.0.txt	
GenMir++	MIRDIP_MICRORNA_E-Version4.0.txt	
MAMI	MIRDIP_MICRORNA_E-Version4.0.txt	
MBStar	https://www.isical.ac.in/~bioinfo_miu/MBStar/MBStar_download20.htm	Refseq to Gene
MicroCosm	multiMIR R package	
miR2Disease	http://www.mir2disease.org	miRtar.txt
MirAncesTar	MIRDIP_MICRORNA_E-Version4.0.txt	
mirBase	MIRDIP_MICRORNA_E-Version4.0.txt	
miRanda	multiMIR R package	
miRBridge	http://mirsystem.cgm.ntu.edu.tw/index.php	mature microRNAs
miRcode	MIRDIP_MICRORNA_E-Version4.0.txt	
mirCoX	MIRDIP_MICRORNA_E-Version4.0.txt	
miRDsnp	http://mirdsnp.ccr.buffalo.edu/download.php	
miRDB	http://mirdb.org	miRDB V6.0 prediction
mirDIP	MIRDIP_MICRORNA_E-Version4.0.txt	score class=E
miRecords†	http://c1.accurascience.com/miRecords/download.php	Homo sapiens
MirGate	http://mirgate.bioinfo.cnio.es/miRGate	REST API
miRMap	https://mirmap.ezlab.org Homo sapiens mirmap201301e	percentile >=80%
MirSNP	MIRDIP_MICRORNA_E-Version4.0.txt	
MirTar	MIRDIP_MICRORNA_E-Version4.0.txt	
miRTar2GO	http://www.mirtar2go.org	highly sensitive
miRTarBase†	multiMIR R package	
mirWalk3	http://mirwalk.umm.uni-heidelberg.de (3'UTR,5'UTR,CDS)	>=0.8
Mirza-G	MIRDIP_MICRORNA_E-Version4.0.txt	
MultiMiTar	https://www.isical.ac.in/~bioinfo_miu/multimitar.htm	score quantile >0.2
OncomiRDB†	oncomirdb.v-1.1-20131217_download.txt	
PACCMIT	https://paccmit.epfl.ch	
PharmacoMir	multiMIR R package	
PITA	https://genie.weizmann.ac.il	ddG =< -10
PolymiRTS†	https://compbio.uthsc.edu/miRSNP/browse_clash.php	
RNA22	https://cm.jefferson.edu/rna22-full-sets-of-predictions	ENSEMBL96 _miRBase22.tar.gz
RPmirDP	https://doi.org/10.5683/SP2/LD8JKJ	score>=0.6
StarBase†	Bash script	>=1 CLIP experiment
TargetRank	http://hollywood.mit.edu/targetrank/	removed 6mer
TargetScan	http://www.targetscan.org/vert_72/	context++ sc. perc >= 70
TargetSpy	http://webclu.bio.wzw.tum.de/targetspy	score>0.99

† databases including validated microRNA targets, MIRDIP_MICRORNA_E-Version4.0.txt has been downloaded on 7/18/2021 from https://ophid.utoronto.ca/mirDIP.

5 Detailed methods

All computational methods in MIO are implemented in Python version 3.8.8 with respective modules such as *scipy* and *sklearn* (https://www.python.org), or the statistical software environment R version 4.1.0 (https://www.R-project.org, R Foundation for Statistical Computing, Vienna, Austria).

Correlation and regularized regression analyses

We use correlation analysis methods as well as regularized regression models to compute the relationship between microRNAs and genes, based on their normalized expression across all selected samples. Correlation is based on Pearson's correlation, Spearman's rank based correlation ρ, and Kendall's τ using the *scipy* module. Correlation analyses based on the non-parametric Hoeffding's independence test (Hoeffding, 1948) and the randomized dependence coefficient (RDC) were directly implemented. In regularized regression models each gene is presented as a linear combination of microRNAs and the coefficients of individual microRNAs in that models can be used as another measure for the association strength (Muniategui et al., 2012). The least absolute shrinkage and selection operator (Lasso) uses L1 regularization (Tibshirani, 1996), the Ridge regression uses L2 regularization (squared coefficient as penalty term to the loss function), whereas in Elastic Net regression a combination of both is used (Zou and Hastie, 2005). This regression models were implemented using the Python *sklearn* module. Each method has different assumptions and advantages and it has been shown that ensemble methods, which integrate different methods, perform better than the individual component methods. Therefore, we used a rank scoring system after Jean-Charles de Borda based on individual rankings for each method (Le *et al.*, 2015; Marbach *et al.*, 2012).
In order to rule out random correlations, a background (null) model distribution of the correlation of a microRNA with 1000 randomly selected genes without evidence of any of the 40 target prediction tools is tested (List et al., 2019).

If the survival meta-data are included, $\log_2$ hazard ratios ($\log_2$ HR) can be computed for each expression ratio of a miRNA and its potential target gene using a Cox proportional hazard model implemented in the *lifeness* Python module. Absolute values of $\log_2$ HR are then also included in the ranking system. The results from correlation analyses can be filtered based on the correlation and logical combination of integrated prediction tools and visualized as interactive microRNA-gene network using Cytoscape with several network layout options. MicroRNAs and genes are represented as nodes that can be colored according to log2 hazard ratio, and edges according to correlation.

MicroRNA targeting a complete pathway or gene set in total (module score)

Of interest is not only which individual genes a microRNA is targeting, but if a pathway or process can be modulated/regulated in total by this microRNA. For this purpose we have adapted a previously suggested score (Buffa *et al.*, 2011), whereby the idea is that expression of genes are weighted based on supporting evidence to be a direct target of the microRNA i.e. number of target prediction tools (n_s) for the respective gene j and the weight p_j=n_s/40. The weighted expression score S for a specific microRNA and a sample (patient) is calculated across all genes in the pathways (gene set) as

$$S = \sum p_j \, x \, e_j / \sum p_j$$

with e_j indicating the expression of the respective gene j. This score is calculated for each patient sample and the association between the expression score and the microRNA expression over all patients is calculated using the Spearman's rank correlation and visualized as scatter plot.

MicroRNAs association with the immunophenoscore or estimated tumor infiltrated immune cells

In order to analyze whether microRNAs are associated with immune scores such as immunophenoscore (IPS) or estimated tumor infiltrated immune cells the same correlation and regularized regression methods as for the calculation of the microRNA-gene correlations are available. For pre-calculation, we have used the R package *immunedeconv* (Sturm *et al.*, 2019) including four tools EPIC (Racle *et al.*, 2017), MCP counter (Becht *et al.*, 2016), quanTIseq (Finotello *et al.*, 2019), xCell (Aran *et al.*, 2017) to estimate infiltrated immune cells in different cancer types from TCGA based on normalized RNA sequencing data (TPM) and implemented in Python the calculation of the immunophenoscore (IPS) using the continuous raw score (averaged z-score) as described in (Charoentong *et al.*, 2017). Results are presented in tabular format and as a heatmap. Individual associations can be displayed as scatter plot.

Identification of prognostic biomarkers and survival analyses

In order to identify prognostic biomarkers MIO implements a feature selection analysis for microRNAs, genes, or ratios of microRNAs and genes using three survival analysis methods (Poelsterl, 2020). This include gradient boosting (using 1000 trees and a learning rate of 0.5), fast survival support vector machines, and a penalized Cox model. In order to obtain the most robust estimator, each model is run several times with different parts of the dataset to avoid over-fitting. For each method the features are ranked according to the average importance of the feature in all partitions and the overall percentage is given, indicating the number of times this feature has appeared in all models with all partitions. For overall ranking of features a scoring system after Jean-Charles de Borda based on individual rankings of each method is provided (Le *et al.*, 2015; Marbach *et al.*, 2012).

Patients can be divided into two groups based on microRNA or gene expression levels using quantile (e.g. median) or optimized dichotomization (with maximal log-rank statistics using R package *survminer*) and Kaplan Meier curves for provided survival data are visualized and the difference is tested with log-rank test and hazard ratio is calculated using the *lifeness* Python module.

Identification of predictive biomarkers and classification analyses

Similar to survival analyses, a feature selection analysis for classification of two groups of patients (e.g. responder versus non-responder of a therapy) including microRNAs, genes, or ratios of microRNAs and genes (representing microRNA-gene network biomarkers) is possible. For this purpose, six different machine learning methods (Hastie *et al.*, 2011) are applied in a cross validation procedure to avoid overfitting. These different methods are implemented in the *sklearn* Python module and includes a random forest classifier (Breiman, 2001) with 300 trees, logistic regression with L2 penalty function and a maximum of 10000 iterations, a Ridge classifier with maximum of 10000 iterations, support vector machines (Vapnik and Chervonenkis, 1974), an Ada boosting classifier (Yoav and Schapire, 1997) with

300 trees, and a bagging classifier (Breiman, 1996) with 300 trees. Some of these tools take advantage of ensemble methods by combining the prediction of several base estimators to improve generalizability or robustness over a single estimator. For each method, the features can be ranked according to the average importance of the feature in all partitions and the overall percentage is given, indicating the number of times this feature has appeared in all models with all partitions.

Based on selected features a classification analysis can be performed by applying the widely used methods random forest (Breiman, 2001), logistic regression, or support vector machines (SVM) (Vapnik and Chervonenkis, 1974) including a k-fold cross validation procedure. The performance of the classifier can be assessed using a receiver operating characteristics curve (ROC) and the respective area under curve (AUC). Ranked feature importance (weights) can be visualized as barplots and separation of samples by the expression of selected features are accessible by principal component analyses. As it is highly recommended to test the classification model also on an independent validation data set, the model and its parameters can be saved and applied to other datasets.

Prediction of microRNAs targeting genes which are synthetic lethal to other candidates

For this type of analysis, we have taken advantage of previous efforts in identifying synthetic lethal gene pairs. The ISLE (identification of clinically relevant synthetic lethality) algorithm (Lee *et al.*, 2018), which is based on data on CRISPR/RNAi *in vitro* screens of cancer cell lines with impact on survival of patients from different TCGA cohorts as well as with phylogenetic evidence was applied and synthetic lethal partner genes were identified (using R code from https://github.com/jooslee/ISLE). Individual candidate genes or genes from a specified gene set (for instance target or essential genes for immunotherapy or targeted therapy) can be selected and by using information of combined microRNA target prediction tools, microRNAs can be identified potentially targeting genes which are synthetic lethal to these candidates. Analysis of potential (off) targets of microRNAs can be performed using the respective feature in MIO for microRNA target prediction.

USE CASES

Use Case S1: MicroRNAs targeting immune modulators including PD-L1

We were interested in finding out which are the most important microRNAs regulating immune-checkpoints in tumor cells. For this purpose, within MIO we employed a correlation analysis for the available lung adenocarcinoma dataset (*TCGA-LUAD*) filtered for primary tumor (*Filter samples to get* correlation: *Group name in metadata for filter=sample_type*, *Group Name in metadata for correlation=PrimaryTumor*) and for the immune checkpoints genes (*Select geneset to analyze=Immune Checkpoints [ICBI]*). MicroRNA target predictions were then based on this correlation analysis (*Use miRNA/gene correlation result=LUAD-COR*), FDR<0.1 (*Maximum Adjust P. Value*=0.1*), with a correlation of microRNA and target gene smaller than -0.3 (*Coefficient equal or lower than* =-0.3, Coefficient equal or higher than* =1.0*) and at least 10 supporting prediction tools (*Filter method to target Databases=OR, selection of all tools, Min db*=10*) for a list of immune checkpoint genes (*Select Genset to Analysis=Immune Checkpoints [ICBI]*).

In Figure S1 resulting microRNAs tageting immune checkpoints are visualized as heatmap and interaction network. Interestingly, miR-200 family members (miR-200a, miR-200b, miR-200c, miR-429, miR-141) are potentially targeting PD-L1 (CD274) as evident also from previous studies (Chen *et al.*, 2014; Grenda *et al.*, 2020).

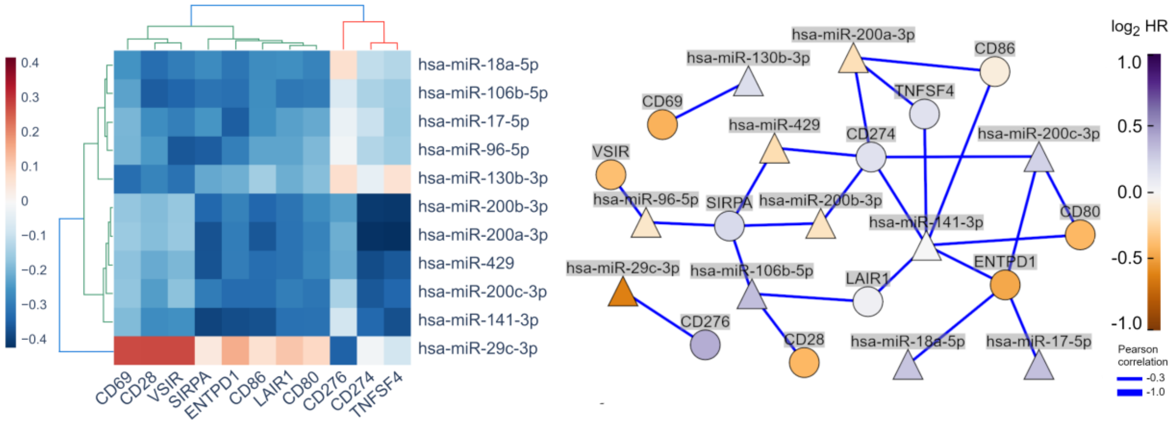

Figure S1: Heatmap of microRNA-immune checkpoint genes correlation (Pearson correlation coefficient <-0.3, at least 10 supporting microRNA target prediction tools) in primary tumors of the LUAD-TCGA data set (left) and respective microRNA gene interaction network (right) (node colors are according to log2-hazard ratio of overall survival with median dichotomization).

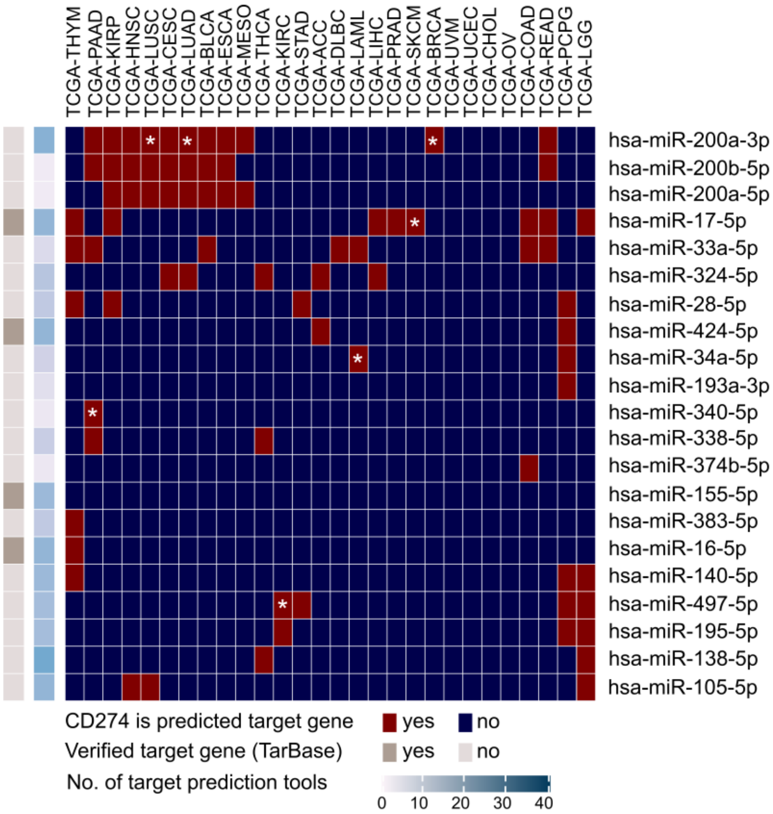

Figure S2: Heatmap of microRNA-immune checkpoint genes targets in various cancer types (* indicating evidence of microRNA targeting PD-L1 in the respective cancer type)

In order to systematically compare microRNAs targeting PD-L1 (CD274) predicted using MIO for microRNAs previously described in (Danbaran *et al.*, 2020; Eichmüller *et al.*, 2017; Incorvaia *et al.*, 2020; Omar *et al.*, 2019) we performed a similar analyses across various cancer types from TCGA (Figure S2). From the 27 described microRNAs, MIO could identify 21. Several microRNAs targeting PD-L1 in specific cancer types could be confirmed such as miR-17 in melanoma (Audrito *et al.*, 2017), miR-34a in AML (Wang *et al.*, 2015), miR-497 in clear cell renal carcinoma (Qu *et al.*, 2019), miR-340 in pancreatic cancer (Dong *et al.*, 2018), and miR-200 in breast cancer (Noman *et al.*, 2017), lung cancer (Chen *et al.*, 2014).

Use Case S2: Prognostic relevant microRNA target gene pairs

There are different types of immune modulators known, which could be stimulatory or inhibitory to the immune response thereby affecting cancer patients overall survival. We are testing which microRNAs are targeting the immune checkpoints and are prognostic in lung adenocarcinoma. One particular analysis possible with MIO is to identify which microRNA/gene ratios are prognostic. We used *MicroRNA/Gene Ratio Feature Selection*, *Select geneset to analyze=Immune Checkpoints [ICBI], Select dataset to analyze=TCGA-LUAD, Group name in metadata=event, Obtain top predictors=50, Number of cross-validation*=3. One of the top candidates with highest feature importance (Cox PH model) was the hsa-miR-150-5p/CD276 ratio (Figure S3 B). Interestingly, we could identify that hsa-miR-150-5p is targeting CD276 (also known as B7-H3) with opposite effect on patient survival outcome as indicated by the network visualization with log2 hazard ratio and in the Kaplan-Meier survival curves with optimized dichotomization (Figure S3A).

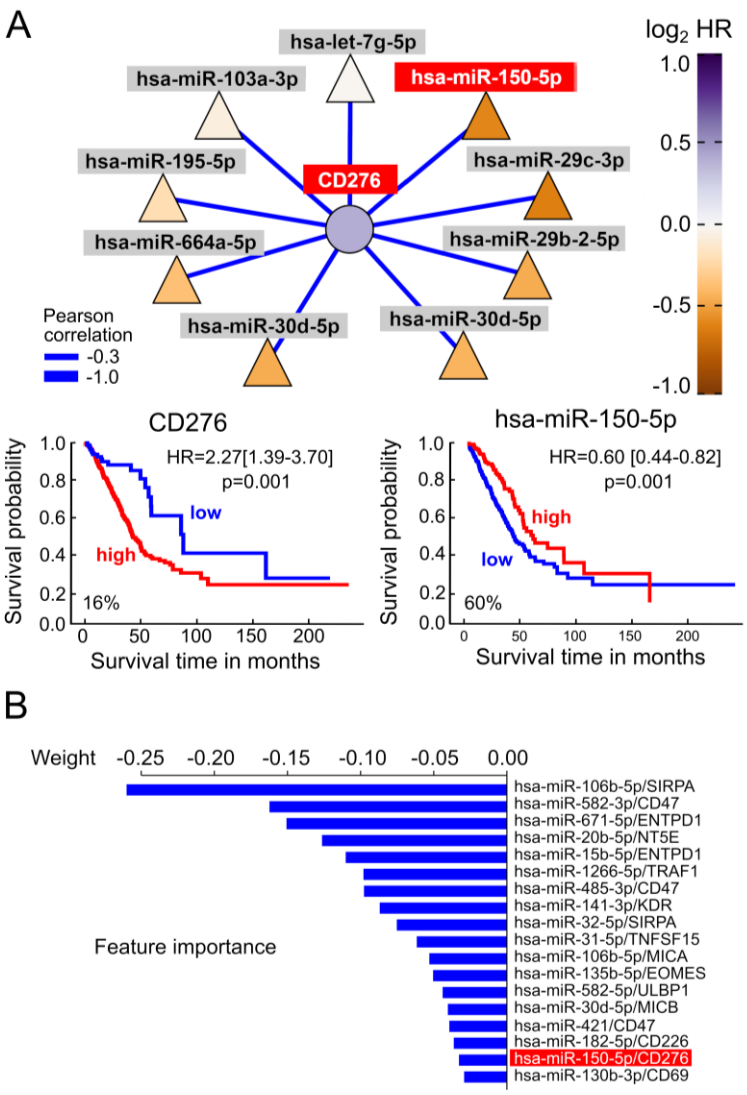

Figure S3: Network visualization of microRNA targeting the immune modulator CD276 and Kaplan-Meier survival curves for hsa-miR-150-5p and CD276 with optimized dichotomization in the lung adenocarcinoma TCGA data set (A). Feature importance for identifying prognostic microRNA/gene ratios (B). Node color represents log2 hazard ratio according to legend to the right calculated between patient groups with high gene or microRNA expression versus low gene or microRNA expression based on median dichotomization.

Use Case S3: Genes involved in antigen processing and presentation targeted by microRNAs

Deficient or down-regulated genes of the antigen processing and presentation machinery (APP) have been associated with response prediction to cancer immunotherapy. In order to study which microRNAs are potentially able to down-regulate not only individual genes but also the complete geneset/pathway we performed a correlation analysis using for each microRNA a weighted expression score calculated based on supporting microRNA target prediction tools for the respective genes. As an example, we used two cohorts from different cancer types from TCGA the bladder cancer (BLCA-TCGA) and the acute myeloid leukemia dataset (LAML-TCGA) to test against the antigen processing and presentation geneset (*Select geneset to analyze=Antigen Processing and Presentation [ImmPort]*). The top microRNAs with most negative correlation with the antigen processing and presentation pathway were filtered for Pearson's correlation coefficient R< -0.4 at an FDR<0.1. Using this settings there are 22 microRNAs in BLCA potentially targeting the APP (Table S6) and 3 microRNAs in AML (Table

S7). In MIO, this association can be also visualized as scatter plot and an respective example is shown in Figure S3.

Table S6: MicroRNAs targeting the antigen processing and presentation pathway in BLCA-TCGA (R < -0.4)

Rank	Gene set	MicroRNA	FDR	R
1	Antigen Processig and Presentation [ImmPort]	hsa-miR-191-3p	<0.001	-0.570
2	Antigen Processig and Presentation [ImmPort]	hsa-miR-200a-3p	<0.001	-0.565
3	Antigen Processig and Presentation [ImmPort]	hsa-miR-429	<0.001	-0.548
4	Antigen Processig and Presentation [ImmPort]	hsa-miR-200b-3p	<0.001	-0.539
5	Antigen Processig and Presentation [ImmPort]	hsa-miR-200b-5p	<0.001	-0.519
6	Antigen Processig and Presentation [ImmPort]	hsa-miR-200a-5p	<0.001	-0.506
7	Antigen Processig and Presentation [ImmPort]	hsa-miR-183-5p	<0.001	-0.499
8	Antigen Processig and Presentation [ImmPort]	hsa-miR-769-5p	<0.001	-0.495
9	Antigen Processig and Presentation [ImmPort]	hsa-miR-191-5p	<0.001	-0.480
10	Antigen Processig and Presentation [ImmPort]	hsa-miR-96-5p	<0.001	-0.472
11	Antigen Processig and Presentation [ImmPort]	hsa-miR-1296-5p	<0.001	-0.460
12	Antigen Processig and Presentation [ImmPort]	hsa-miR-93-5p	<0.001	-0.458
13	Antigen Processig and Presentation [ImmPort]	hsa-miR-425-3p	<0.001	-0.457
14	Antigen Processig and Presentation [ImmPort]	hsa-miR-182-5p	<0.001	-0.456
15	Antigen Processig and Presentation [ImmPort]	hsa-miR-324-3p	<0.001	-0.452
16	Antigen Processig and Presentation [ImmPort]	hsa-miR-93-3p	<0.001	-0.448
17	Antigen Processig and Presentation [ImmPort]	hsa-miR-151a-3p	<0.001	-0.446
18	Antigen Processig and Presentation [ImmPort]	hsa-miR-940	<0.001	-0.444
19	Antigen Processig and Presentation [ImmPort]	hsa-miR-301b-3p	<0.001	-0.442
20	Antigen Processig and Presentation [ImmPort]	hsa-miR-106b-5p	<0.001	-0.438
21	Antigen Processig and Presentation [ImmPort]	hsa-miR-141-3p	<0.001	-0.430
22	Antigen Processig and Presentation [ImmPort]	hsa-miR-7706	<0.001	-0.418

Table S7: MicroRNAs targeting the antigen processing and presentation pathway in LAML-TCGA (R < -0.45)

Rank	Gene set	MicroRNA	FDR	R
1	Antigen Processig and Presentation [ImmPort]	hsa-miR-181d-5p	< 0.0001	-0.498
2	Antigen Processig and Presentation [ImmPort]	hsa-miR-181c-5p	< 0.0001	-0.456
3	Antigen Processig and Presentation [ImmPort]	hsa-miR-181b-5p	< 0.0001	-0.450

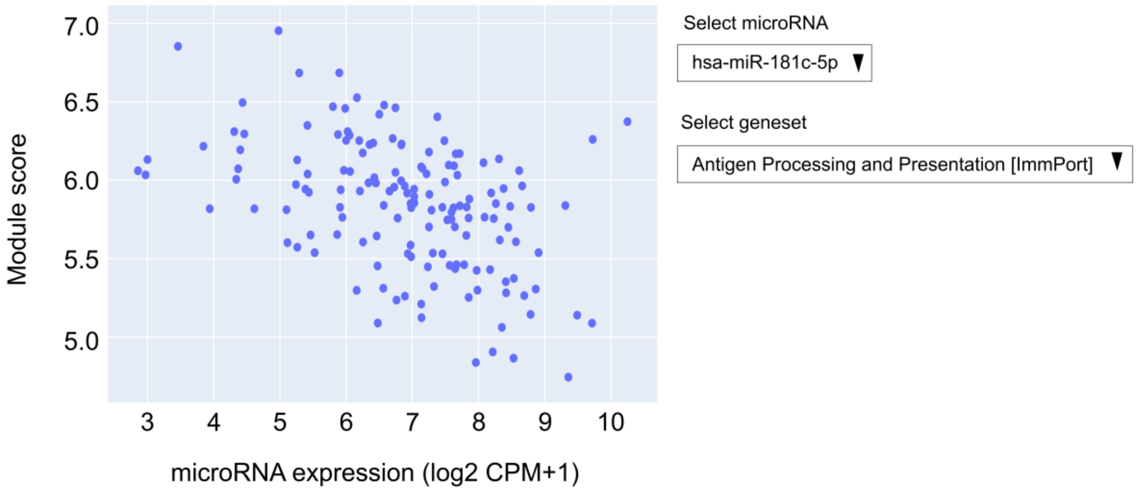

Figure S4: Scatterplot showing the weighted score (module score) for the antigen processing and presentation gene set for each patient is negatively associated with the expression of hsa-miR-181c-5p in the LAML-TCGA dataset.

These two examples show that microRNAs targeting APP can vary depending on the cancer type. In bladder cancer, members of the miR-200 family of microRNAs are among the top candidates. In acute myeloid leukemia, miR-181 family members potentially target APP, which may be of particular interest for immunotherapy of NPM1-mutated AML, as the mutant NPM1 protein has been identified as the source of most immunogenic epitopes (Forghieri et al., 2021). Moreover, the miR-181 family members have been described to be directly involved in myeloid differentiation and AML (Su et al., 2015).

Use case S4: Identifying a microRNA signature predictive for microsatellite instable colorectal cancer samples.

Microsatellite instability (MSI) in colorectal cancer - often associated with deficient DNA mismatch repair pathway genes and a high mutational burden – has been shown to be predictive for immune checkpoint inhibitor therapy (Dudley *et al.*, 2016; Le *et al.*, 2017). We have also previously demonstrated that the MSI molecular subtype in colorectal cancer, in particular, is associated with an immunophenotype that includes a variety of tumor-infiltrating lymphocytes (TILs) (Angelova et al., 2015). Our aim was to identify a microRNA signature that predicts microsatellite high status (MSI-H) compared to microsatellite low/microsatellite stable (MSI-L/MSS) status in colorectal cancer to learn which patients might benefit from immune checkpoint blocker therapy (e.g., anti-PD-1 or anti-PD-L1). Therefore, we retrieved data from the TCGA for a combined colon and rectal cancer cohort (CRC-TCGA) (Cancer Genome Atlas Research Network, 2012b) including 572 patients with primary tumor and another cohort of 105 colon cancer patients (CPTAC-2) (Vasaikar *et al.*, 2019) for validation. The microsatellite instability status according to the Mantis MSI score was retrieved from cBioPortal (https://www.cbioportal.org). Based on the TCGA-CRC cohort we performed a feature selection procedure in order to obtain the 25 most informative microRNAs in separation of MSI-H tumors (with settings in MIO: *Obtain top predictors=25*, *Number of cross-validation=3*, *Select features=miRNAs*, *Group name in metadata=MSI*, *Select dataset to analyze=TCGA-CRC*). The obtained most predictive microRNAs and their frequency included in the top candidates in each fold of the seven classification methods are visualized as heatmap in Figure S5A. Based on the expression of these 25 top microRNA candidates a principal component analysis (PCA) was performed in MIO and the projection to the first three components were visualized to indicate how the two groups can be separated (Figure S5B).

Including the identified microRNAs (which in MIO can be saved as a microRNA set) a classification analysis was performed on the TCGA-CRC data as a training set using a logistic regression model and 10-fold cross validation to separate the MSI samples (*Select data to analyze=TCGA CRC, Select Models=Logistic Regression, Number of cross validation=10, Group name in metadata=MSI, Select miRNAset to analyze=name as saved after feature selection).*

In the TCGA-CRC training set the MSI groups could be separated with an area under receiver operating characteristics (ROC) curve of 0.908 and mean accuracy over 10-fold cross validation of 0.962 (Figure S6). Feature importance (weights) of the individual microRNAs in the logistic regression model are shown in Figure S7. The trained model can be saved in MIO and the performance of this model can then be tested in the validation cohort. Ultimately, the MSI groups were also well separated in the validation cohort as evident from the ROC curve (AUC=0.860) and PCA of the expression of these microRNAs (Figure S8).

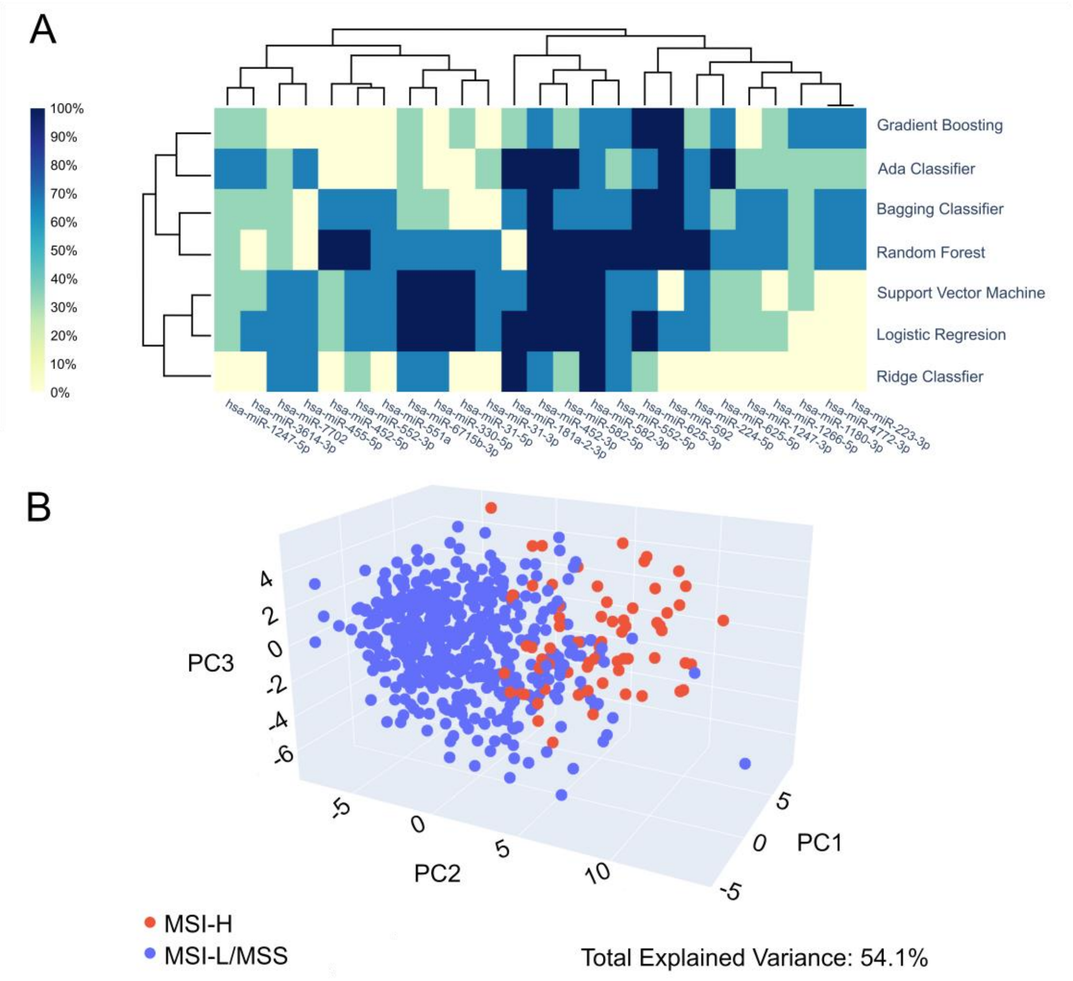

Figure S5: Results from feature selection showing the 25 most informative microRNAs in separating microsatellite instable colorectal cancer patients including a heatmap of the frequency of each microRNAs is included in the top candidates in each fold for the seven classification methods (A) and principal component analyses (PCA) based on the expression of these 25 microRNAs (B).

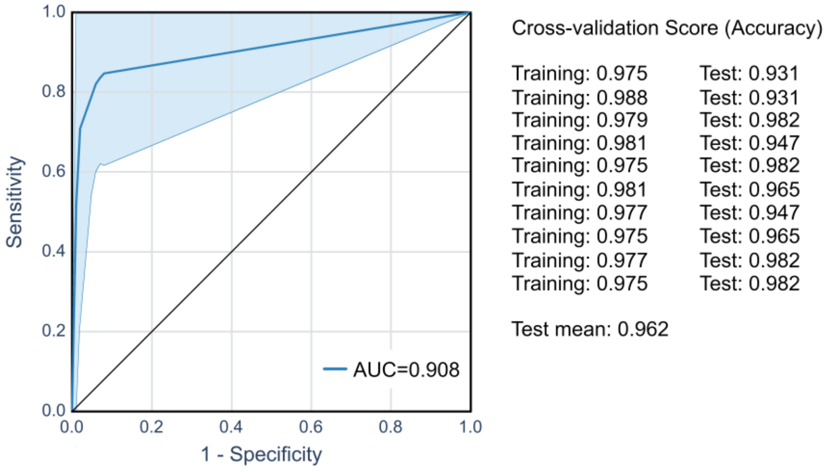

Figure S6: Performance of the 25 microRNA logistic regression model in 10-fold cross validation receiver operating characteristics (ROC) curve with bootstrap confidence interval and accuracy of training and test for each fold in the TCGA-CRC cohort.

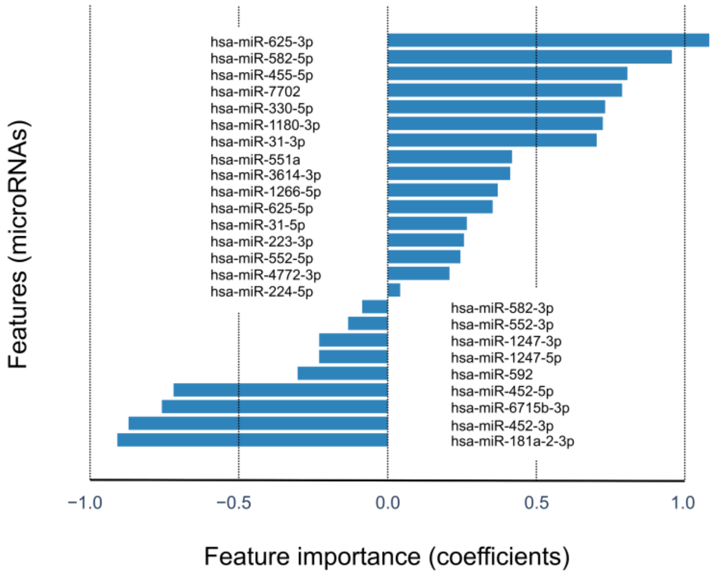

Figure S7: Feature importance (weights) of the individual microRNAs in the logistic regression model.

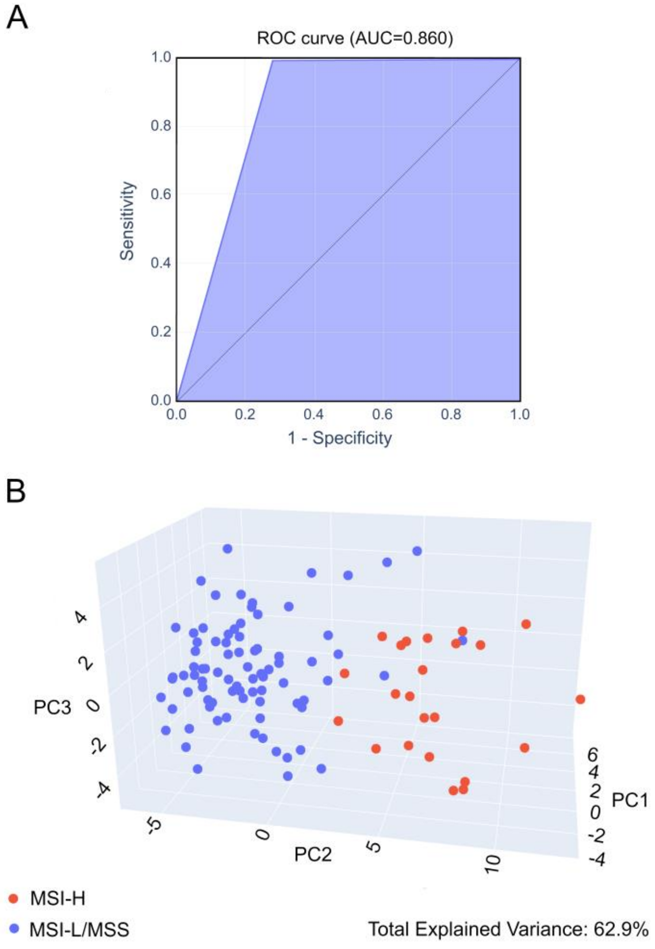

Figure S8: Performance of the 25 microRNA logistic regression model (trained with the TCGA CRC data) in the 105 colon cancer patients of the validation cohort (CPTAC-2) including ROC curve (A) and principal component analyses (PCA) of the expression of the 25 microRNAs separating MSI-H colon cancer patients (B).

Use Case S5: MicroRNA target genes synthetic lethal to immune (therapy) essential genes

Using genome-scale CRISPR–Cas9 library Patel et al. profiled genes whose loss in tumor cells impaired the effector function of CD8+ T cells (Patel *et al.*, 2017). In order to identify synthetic lethal partner genes in tumor cells we have taken advantage of previous efforts and used the ISLE algorithm for calculation (Lee *et al.*, 2018), which is available within MIO. We were interested in identifying microRNAs targeting genes which are synthetic lethal to immune (therapy) essential genes. With regard to combination immunotherapy, either the immunoessential gene is highly expressed/not mutated and tumor cells may be targeted by immunotherapy, or in the case of a low expressed/mutated immune essential gene, targeting of its synthetic lethal gene by e.g. microRNAs could be detrimental to tumor cells and reveal therapeutic vulnerabilities. In MIO we used the option *Target Prediction, miRNA Synthetic Lethal Prediction, Select geneset to analyze=Immune essential genes [Patel], Filter method to target Databases=OR, select all prediction tools, Min db*=25.* In order to provide robust target predictions and to limit the possible targets to a reasonable number we have chosen that microRNA targets are supported by at least 25 of the 40 target prediction tools. This resulted in 321 interactions of microRNAs and synthetic lethal partners of immune essential genes. For clarity, we summarized only the top 25 interactions (supported by 28 target prediction tools) in Table S8 and visualized them as a network (Figure S9). As microRNAs are targeting also other genes (off targets) MIO provides the possibility to identify all targets for individual microRNAs (*miRNA/Gene Target Prediction*). In addition, MIO can perform an overrepresentation analysis for microRNAs based on the number of synthetic lethal target genes compared to all potential target genes (Table S9).

Table S8: MicroRNAs targeting genes, which are synthetic lethal to immune therapy essential genes (Query) supported by at least 28 microRNA target prediction tools.

Query	Synthetic Lethal	MicroRNA	No. prediction tools
RECQL4	BNIP2	hsa-miR-20b-5p	30
MED23	HOXC8	hsa-miR-196a-5p	30
PPARD	ZFX	hsa-miR-144-3p	29
SOX10	ATAD2	hsa-miR-20b-5p	29
RECQL4	BACH1	hsa-miR-155-5p	29
RECQL4	BNIP2	hsa-miR-106a-5p	29
TMEM209	NR4A3	hsa-miR-106a-5p	29
TMEM209	NR4A3	hsa-miR-17-5p	29
TMEM209	NR4A3	hsa-miR-20b-5p	29
AURKA	CNOT8	hsa-miR-7-5p	28
BMP8A	RTN4	hsa-miR-148b-3p	28
MED19	RTN4	hsa-miR-148b-3p	28
MPG	RTN4	hsa-miR-148b-3p	28
SPDEF	RTN4	hsa-miR-148b-3p	28
MYC	PRDM2	hsa-miR-211-5p	28
SV2B	TFG	hsa-miR-221-3p	28
RECQL4	BACH1	hsa-miR-98-5p	28
RECQL4	BNIP2	hsa-miR-106b-5p	28
HGSNAT	DDIT4	hsa-miR-22-3p	28
TLR5	EIF4EBP1	hsa-miR-138-5p	28
MED19	HOXA7	hsa-miR-196a-5p	28
MED23	HOXC8	hsa-miR-196b-5p	28
TMEM209	NR4A3	hsa-miR-106b-5p	28
SLC1A5	PLSCR4	hsa-miR-16-5p	28
ZNF433	SNX17	hsa-miR-19a-3p	28

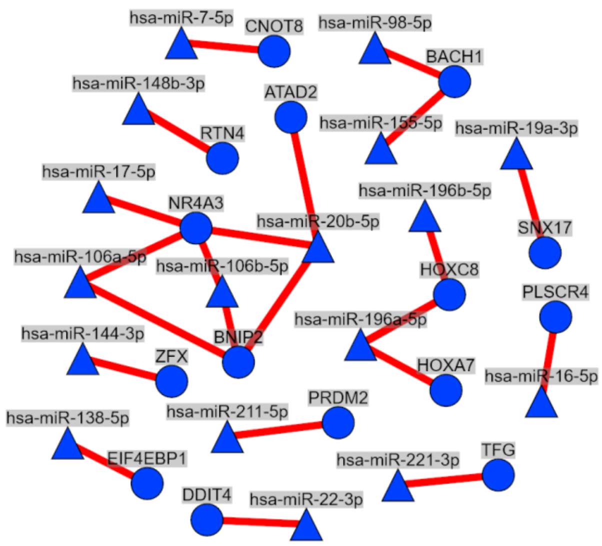

Figure S9: Interaction network of microRNAs targeting genes, which are synthetic lethal to immune therapy essential genes supported by at least 28 microRNA target prediction tools.

Table S9: Overrepresentation of microRNA targeting synthetic lethal partners compared to all targets of the respective microRNA (number of targets are supported by at least 25 microRNA target prediction tools)

MicroRNA	No. targets	Expected number	Fold Enrichment	P	FDR
hsa-miR-20b-5p	18	9.96	1.82	0.02	0.48
hsa-miR-98-5p	4	1.37	2.94	0.06	0.48
hsa-miR-106a-5p	11	6.12	1.81	0.07	0.48
hsa-miR-196b-5p	3	0.96	3.13	0.09	0.48
hsa-miR-17-5p	11	6.22	1.78	0.10	0.48
hsa-miR-221-3p	4	1.92	2.09	0.14	0.51
hsa-miR-196a-5p	2	0.81	2.47	0.21	0.59
hsa-miR-155-5p	2	0.96	2.08	0.27	0.62
hsa-miR-106b-5p	6	3.89	1.54	0.30	0.62
hsa-miR-7-5p	4	2.33	1.72	0.30	0.62
hsa-miR-138-5p	2	1.21	1.65	0.36	0.63
hsa-miR-211-5p	1	0.61	1.65	0.47	0.72
hsa-miR-22-3p	2	1.62	1.24	0.68	0.86
hsa-miR-19a-3p	2	1.87	1.07	0.71	0.87
hsa-miR-148b-3p	3	2.38	1.26	0.73	0.88
hsa-miR-144-3p	1	1.42	0.71	1.00	1.00
hsa-miR-16-5p	3	3.29	0.91	1.00	1.00

Use Case S6: MicroRNAs associated with the immunophenoscore (IPS) and estimated infiltrated immune cells in ovarian cancer (TCGA-OV).

We are interested whether microRNAs are associated with the Immunophenoscore (IPS) or estimated infiltration of immune cells in ovarian cancer (TCGA-OV). Therefore, we performed *miRNA/Immunophenoscore correlation* analysis and *Select dataset to analyze: TCGA-OV and Select the coefficient for applying the filters: Pearson(R), Maximum adjust P.value*:0.1, Coefficient equal or lower than*:-0.3*, *Coefficient equal or higher than*:1.0*). These settings resulted in one microRNA, hsa-miR-223-3p, with the respective scatterplot (Figure S10).

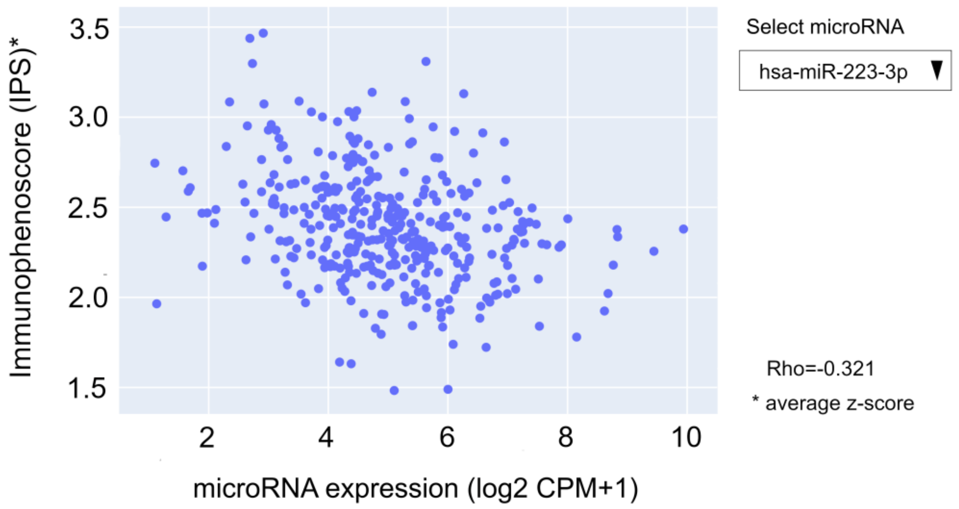

Figure S10: Association of microRNAs with immunophenoscore (IPS) in ovarian cancer (TCGA-OV).

Interestingly hsa-miR-223-3p has been shown to target PARP1 which is involved in homologous recombination repair in ovarian cancer (Srinivasan *et al.*, 2019). PARP inhibition therapy is approved in ovarian cancer and probably could impact the immune environment and immune therapy response via the STING pathway (Ding *et al.*, 2018). Since we could observe an association of hsa-miR-223-3p with the immunophenoscore, we were also interested whether there is an association of microRNAs and in particular hsa-miR-223-3p with decreased estimated immune cell infiltration. Thus, we performed *miRNA/Immune Cell Infiltration Correlation* analyses, *Select dataset to analyze: TCGA-OV,* and selected *QUANTISEQ (B cell, Macrophage M1, Macrophage M2, Monocyte, Neutrophil, NK cell, T cell CD4+ (non-regulatory), T cell CD8+, T cell regulatory (Tregs), Myeolid dendritic cells).* Results of selected examples are summarized in Table S10.

Table S10: MicroRNAs associated with selected estimated immune cell infiltration (quanTIseq)

Gene set	MicroRNA	FDR	Rho
QUANTISEQ T cell CD8+	hsa-miR-708-3p	0.010	-0.287
QUANTISEQ T cell CD4+ (non-regulatory)	hsa-miR-20a-5p	0.007	-0.296
QUANTISEQ T cell CD8+	hsa-miR-223-3p	0.752	0.050
QUANTISEQ Macrophage M2	hsa-miR-223-3p	<0.001	0.325
QUANTISEQ T cell regulatory	hsa-miR-223-3p	<0.001	0.412

Although hsa-miR-223-3p was not significantly associated with estimated CD8+ T cells it shows a positive correlation with a suppressive immune environment (e.g. Tregs).

In summary, a combination of the presented use cases may help to develop new hypotheses in immuno-oncology or identify new targets for cancer immunotherapy.

REFERENCES

Agarwal,V. et al. (2015) Predicting effective microRNA target sites in mammalian mRNAs. Elife, 4.

Ahadi,A. et al. (2017) miRTar2GO: a novel rule-based model learning method for cell line specific microRNA target prediction that integrates Ago2 CLIP-Seq and validated microRNA-target interaction data. Nucleic Acids Res, 45, e42.

Akbani,R. et al. (2015) Genomic Classification of Cutaneous Melanoma. Cell, 161, 1681–1696.

Andrés-León,E. et al. (2015) miRGate: a curated database of human, mouse and rat miRNA-mRNA targets. Database (Oxford), 2015, bav035.

Aran,D. et al. (2017) xCell: digitally portraying the tissue cellular heterogeneity landscape. Genome Biol, 18, 220.

Audrito,V. et al. (2017) PD-L1 up-regulation in melanoma increases disease aggressiveness and is mediated through miR-17-5p. Oncotarget, 8, 15894–15911.

Auslander,N. et al. (2018) Robust prediction of response to immune checkpoint blockade therapy in metastatic melanoma. Nat. Med., 24, 1545–1549.

Ayers,M. et al. (2017) IFN-γ-related mRNA profile predicts clinical response to PD-1 blockade. J Clin Invest, 127, 2930–2940.

Bagaev,A. et al. (2021) Conserved pan-cancer microenvironment subtypes predict response to immunotherapy. Cancer Cell, 39, 845-865.e7.

Bandyopadhyay,S. et al. (2015) MBSTAR: multiple instance learning for predicting specific functional binding sites in microRNA targets. Sci Rep, 5, 8004.

Barry,K.C. et al. (2018) A natural killer-dendritic cell axis defines checkpoint therapy-responsive tumor microenvironments. Nat Med, 24, 1178–1191.

Becht,E. et al. (2016) Estimating the population abundance of tissue-infiltrating immune and stromal cell populations using gene expression. Genome Biol, 17, 218.

Bhattacharya,A. et al. (2014) PolymiRTS Database 3.0: linking polymorphisms in microRNAs and their target sites with human diseases and biological pathways. Nucleic Acids Res, 42, D86-91.

Bhattacharya,S. et al. (2014) ImmPort: disseminating data to the public for the future of immunology. Immunol Res, 58, 234–239.

Bindea,G. et al. (2013) Spatiotemporal dynamics of intratumoral immune cells reveal the immune landscape in human cancer. Immunity, 39, 782–795.

Breiman,L. (1996) Bagging predictors. Machine Learning, 24, 123–140.

Breiman,L. (2001) Random forests. Machine Learning, 45, 5–32.

Bruno,A.E. et al. (2012) miRdSNP: a database of disease-associated SNPs and microRNA target sites on 3’UTRs of human genes. BMC Genomics, 13, 44.

Buffa,F.M. et al. (2011) microRNA-associated progression pathways and potential therapeutic targets identified by integrated mRNA and microRNA expression profiling in breast cancer. Cancer Res, 71, 5635–5645.

Cabrita,R. et al. (2020) Tertiary lymphoid structures improve immunotherapy and survival in melanoma. Nature, 577, 561–565.

Cancer Genome Atlas Research Network (2017a) Comprehensive and Integrated Genomic Characterization of Adult Soft Tissue Sarcomas. Cell, 171, 950-965.e28.

Cancer Genome Atlas Research Network (2017b) Comprehensive and Integrative Genomic Characterization of Hepatocellular Carcinoma. Cell, 169, 1327-1341.e23.

Cancer Genome Atlas Research Network (2008) Comprehensive genomic characterization defines human glioblastoma genes and core pathways. Nature, 455, 1061–1068.

Cancer Genome Atlas Research Network (2015a) Comprehensive genomic characterization of head and neck squamous cell carcinomas. Nature, 517, 576–582.

Cancer Genome Atlas Research Network (2012a) Comprehensive genomic characterization of squamous cell lung cancers. Nature, 489, 519–525.

Cancer Genome Atlas Research Network (2015b) Comprehensive, Integrative Genomic Analysis of Diffuse Lower-Grade Gliomas. N Engl J Med, 372, 2481–2498.

Cancer Genome Atlas Research Network (2013a) Comprehensive molecular characterization of clear cell renal cell carcinoma. Nature, 499, 43–49.

Cancer Genome Atlas Research Network (2014a) Comprehensive molecular characterization of gastric adenocarcinoma. Nature, 513, 202–209.

Cancer Genome Atlas Research Network (2012b) Comprehensive molecular characterization of human colon and rectal cancer. Nature, 487, 330–337.

Cancer Genome Atlas Research Network (2016) Comprehensive Molecular Characterization of Papillary Renal-Cell Carcinoma. N Engl J Med, 374, 135–145.

Cancer Genome Atlas Research Network (2012c) Comprehensive molecular portraits of human breast tumours. Nature, 490, 61–70.

Cancer Genome Atlas Research Network (2014b) Comprehensive molecular profiling of lung adenocarcinoma. Nature, 511, 543–550.

Cancer Genome Atlas Research Network et al. (2013) Genomic and epigenomic landscapes of adult de novo acute myeloid leukemia. N Engl J Med, 368, 2059–2074.

Cancer Genome Atlas Research Network (2011) Integrated genomic analyses of ovarian carcinoma. Nature, 474, 609–615.

Cancer Genome Atlas Research Network (2017c) Integrated genomic and molecular characterization of cervical cancer. Nature, 543, 378–384.

Cancer Genome Atlas Research Network (2013b) Integrated genomic characterization of endometrial carcinoma. Nature, 497, 67–73.

Cancer Genome Atlas Research Network (2017d) Integrated genomic characterization of oesophageal carcinoma. Nature, 541, 169–175.

Cancer Genome Atlas Research Network (2017e) Integrated Genomic Characterization of Pancreatic Ductal Adenocarcinoma. Cancer Cell, 32, 185-203.e13.

Cancer Genome Atlas Research Network (2014c) Integrated genomic characterization of papillary thyroid carcinoma. Cell, 159, 676–690.

Cancer Genome Atlas Research Network (2015c) The Molecular Taxonomy of Primary Prostate Cancer. Cell, 163, 1011–1025.

Charoentong,P. et al. (2017) Pan-cancer Immunogenomic Analyses Reveal Genotype-Immunophenotype Relationships and Predictors of Response to Checkpoint Blockade. Cell Rep, 18, 248–262.

Chen,D.-T. et al. (2012) Complementary strand microRNAs mediate acquisition of metastatic potential in colonic adenocarcinoma. J Gastrointest Surg, 16, 905–912; discussion 912-913.

Chen,Limo et al. (2014) Metastasis is regulated via microRNA-200/ZEB1 axis control of tumour cell PD-L1 expression and intratumoral immunosuppression. Nat Commun, 5, 5241.

Chen,Y. and Wang,X. (2020) miRDB: an online database for prediction of functional microRNA targets. Nucleic Acids Res, 48, D127–D131.

Cherniack,A.D. et al. (2017) Integrated Molecular Characterization of Uterine Carcinosarcoma. Cancer Cell, 31, 411–423.

Chiu,H.-S. et al. (2015) Cupid: simultaneous reconstruction of microRNA-target and ceRNA networks. Genome Res, 25, 257–267.

Cho,S. et al. (2020) MicroRNAs and Their Targetomes in Tumor-Immune Communication. Cancers (Basel), 12, E2025.

Cortez,M.A. et al. (2019) Role of miRNAs in immune responses and immunotherapy in cancer. Genes Chromosomes Cancer, 58, 244–253.

Danaher,P. et al. (2018) Pan-cancer adaptive immune resistance as defined by the Tumor Inflammation Signature (TIS): results from The Cancer Genome Atlas (TCGA). J Immunother Cancer, 6, 63.

Danbaran,G.R. et al. (2020) How microRNAs affect the PD-L1 and its synthetic pathway in cancer. Int Immunopharmacol, 84, 106594.

Davis,C.F. et al. (2014) The Somatic Genomic Landscape of Chromophobe Renal Cell Carcinoma. Cancer Cell, 26, 319–330.

Ding,L. et al. (2018) PARP Inhibition Elicits STING-Dependent Antitumor Immunity in Brca1-Deficient Ovarian Cancer. Cell Reports, 25, 2972-2980.e5.

Dong,P. et al. (2018) Control of PD-L1 expression by miR-140/142/340/383 and oncogenic activation of the OCT4-miR-18a pathway in cervical cancer. Oncogene, 37, 5257–5268.

Dudley,J.C. et al. (2016) Microsatellite Instability as a Biomarker for PD-1 Blockade. Clin Cancer Res, 22, 813–820.

Eichmüller,S.B. et al. (2017) Immune Modulatory microRNAs Involved in Tumor Attack and Tumor Immune Escape. J Natl Cancer Inst, 109.

Farshidfar,F. et al. (2017) Integrative Genomic Analysis of Cholangiocarcinoma Identifies Distinct IDH-Mutant Molecular Profiles. Cell Reports, 18, 2780–2794.
Finotello,F. et al. (2019) Molecular and pharmacological modulators of the tumor immune contexture revealed by deconvolution of RNA-seq data. Genome Med, 11, 34.
Fishbein,L. et al. (2017) Comprehensive Molecular Characterization of Pheochromocytoma and Paraganglioma. Cancer Cell, 31, 181–193.
Forghieri,F. et al. (2021) Neoantigen-Specific T-Cell Immune Responses: The Paradigm of NPM1-Mutated Acute Myeloid Leukemia. Int J Mol Sci, 22, 9159.
Gaidatzis,D. et al. (2007) Inference of miRNA targets using evolutionary conservation and pathway analysis. BMC Bioinformatics, 8, 69.
Gennarino,V.A. et al. (2012) Identification of microRNA-regulated gene networks by expression analysis of target genes. Genome Res, 22, 1163–1172.
Giles,C.B. et al. (2013) mirCoX: a database of miRNA-mRNA expression correlations derived from RNA-seq meta-analysis. BMC Bioinformatics, 14 Suppl 14, S17.
Grenda,A. et al. (2020) Tissue MicroRNA Expression as a Predictor of Response to Immunotherapy in NSCLC Patients. Front Oncol, 10, 563613.
Griffiths-Jones,S. et al. (2008) miRBase: tools for microRNA genomics. Nucleic Acids Res, 36, D154-158.
Gumienny,R. and Zavolan,M. (2015) Accurate transcriptome-wide prediction of microRNA targets and small interfering RNA off-targets with MIRZA-G. Nucleic Acids Res, 43, 1380–1391.
Hastie,T. et al. (2011) The Elements of Statistical Learning Second Edition. Springer.
Higgs,B.W. et al. (2018) Interferon Gamma Messenger RNA Signature in Tumor Biopsies Predicts Outcomes in Patients with Non-Small Cell Lung Carcinoma or Urothelial Cancer Treated with Durvalumab. Clin Cancer Res, 24, 3857–3866.
Hirschberger,S. et al. (2018) MiRNAs: dynamic regulators of immune cell functions in inflammation and cancer. Cancer Lett, 431, 11–21.
Hmeljak,J. et al. (2018) Integrative Molecular Characterization of Malignant Pleural Mesothelioma. Cancer Discov, 8, 1548–1565.
Hoeffding,W. (1948) A non-parametric test of independence. Ann Math Stat, 19, 546–557.
Hsu,J.B.-K. et al. (2011) miRTar: an integrated system for identifying miRNA-target interactions in human. BMC Bioinformatics, 12, 300.
Huang,H.-Y. et al. (2020) miRTarBase 2020: updates to the experimentally validated microRNA-target interaction database. Nucleic Acids Res, 48, D148–D154.
Huang,J.C. et al. (2007) Using expression profiling data to identify human microRNA targets. Nat Methods, 4, 1045–1049.
Incorvaia,L. et al. (2020) A ‘Lymphocyte MicroRNA Signature’ as Predictive Biomarker of Immunotherapy Response and Plasma PD-1/PD-L1 Expression Levels in Patients with Metastatic Renal Cell Carcinoma: Pointing towards Epigenetic Reprogramming. Cancers (Basel), 12, E3396.
Jeggari,A. et al. (2012) miRcode: a map of putative microRNA target sites in the long non-coding transcriptome. Bioinformatics, 28, 2062–2063.
Jerby-Arnon,L. et al. (2018) A Cancer Cell Program Promotes T Cell Exclusion and Resistance to Checkpoint Blockade. Cell, 175, 984-997.e24.
Jiang,P. et al. (2018) Signatures of T cell dysfunction and exclusion predict cancer immunotherapy response. Nat. Med., 24, 1550–1558.
Jiang,Q. et al. (2009) miR2Disease: a manually curated database for microRNA deregulation in human disease. Nucleic Acids Res, 37, D98-104.
John,B. et al. (2004) Human MicroRNA targets. PLoS Biol, 2, e363.
Karagkouni,D. et al. (2018) DIANA-TarBase v8: a decade-long collection of experimentally supported miRNA-gene interactions. Nucleic Acids Res, 46, D239–D245.
Kertesz,M. et al. (2007) The role of site accessibility in microRNA target recognition. Nat Genet, 39, 1278–1284.
Knijnenburg,T.A. et al. (2018) Genomic and Molecular Landscape of DNA Damage Repair Deficiency across The Cancer Genome Atlas. Cell Rep, 23, 239-254.e6.
Kozomara,A. et al. (2019) miRBase: from microRNA sequences to function. Nucleic Acids Res, 47, D155–D162.
Kozomara,A. and Griffiths-Jones,S. (2014) miRBase: annotating high confidence microRNAs using deep sequencing data. Nucleic Acids Res, 42, D68-73.

Kyrollos,D.G. et al. (2020) RPmirDIP: Reciprocal Perspective improves miRNA targeting prediction. Sci Rep, 10, 11770.
Law,C.W. et al. (2014) voom: precision weights unlock linear model analysis tools for RNA-seq read counts. Genome Biol, 15, R29.
Le,D.T. et al. (2017) Mismatch repair deficiency predicts response of solid tumors to PD-1 blockade. Science, 357, 409–413.
Le,T.D. et al. (2015) Ensemble Methods for MiRNA Target Prediction from Expression Data. PLoS ONE, 10, e0131627.
Leclercq,M. et al. (2017) Prediction of human miRNA target genes using computationally reconstructed ancestral mammalian sequences. Nucleic Acids Res, 45, 556–566.
Lee,J.S. et al. (2018) Harnessing synthetic lethality to predict the response to cancer treatment. Nat Commun, 9, 2546.
Li,J.-H. et al. (2014) starBase v2.0: decoding miRNA-ceRNA, miRNA-ncRNA and protein-RNA interaction networks from large-scale CLIP-Seq data. Nucleic Acids Res, 42, D92-97.
Li,R. et al. (2018) GDCRNATools: an R/Bioconductor package for integrative analysis of lncRNA, miRNA and mRNA data in GDC. Bioinformatics, 34, 2515–2517.
Liberzon,A. et al. (2015) The Molecular Signatures Database (MSigDB) hallmark gene set collection. Cell Syst, 1, 417–425.
List,M. et al. (2019) Large-scale inference of competing endogenous RNA networks with sparse partial correlation. Bioinformatics, 35, i596–i604.
Litchfield,K. et al. (2021) Meta-analysis of tumor- and T cell-intrinsic mechanisms of sensitization to checkpoint inhibition. Cell, 184, 596-614.e14.
Liu,C. et al. (2012) MirSNP, a database of polymorphisms altering miRNA target sites, identifies miRNA-related SNPs in GWAS SNPs and eQTLs. BMC Genomics, 13, 661.
Loher,P. and Rigoutsos,I. (2012) Interactive exploration of RNA22 microRNA target predictions. Bioinformatics, 28, 3322–3323.
Lone,S.N. et al. (2021) miRNAs as novel immunoregulators in cancer. Semin Cell Dev Biol, S1084-9521(21)00086–0.
Marbach,D. et al. (2012) Wisdom of crowds for robust gene network inference. Nat Methods, 9, 796–804.
Marín,R.M. et al. (2012) Analysis of the accessibility of CLIP bound sites reveals that nucleation of the miRNA:mRNA pairing occurs preferentially at the 3'-end of the seed match. RNA, 18, 1760–1770.
Mitra,R. and Bandyopadhyay,S. (2011) MultiMiTar: a novel multi objective optimization based miRNA-target prediction method. PLoS One, 6, e24583.
Nielsen,C.B. et al. (2007) Determinants of targeting by endogenous and exogenous microRNAs and siRNAs. RNA, 13, 1894–1910.
Nirmal,A.J. et al. (2018) Immune Cell Gene Signatures for Profiling the Microenvironment of Solid Tumors. Cancer Immunol Res, 6, 1388–1400.
Nishida,N. et al. (2012) Microarray analysis of colorectal cancer stromal tissue reveals upregulation of two oncogenic miRNA clusters. Clin Cancer Res, 18, 3054–3070.
Noman,M.Z. et al. (2017) The immune checkpoint ligand PD-L1 is upregulated in EMT-activated human breast cancer cells by a mechanism involving ZEB-1 and miR-200. Oncoimmunology, 6, e1263412.
Ock,C.-Y. et al. (2017) Genomic landscape associated with potential response to anti-CTLA-4 treatment in cancers. Nat Commun, 8, 1050.
Omar,H.A. et al. (2019) Immunomodulatory MicroRNAs in cancer: targeting immune checkpoints and the tumor microenvironment. FEBS J, 286, 3540–3557.
Paraskevopoulou,M.D. et al. (2013) DIANA-microT web server v5.0: service integration into miRNA functional analysis workflows. Nucleic Acids Res, 41, W169-173.
Patel,S.J. et al. (2017) Identification of essential genes for cancer immunotherapy. Nature, 548, 537–542.
Poelsterl,S. (2020) scikit-survival: A Library for Time-to-Event Analysis Built on Top of scikit-learn. JMLR, 21, 1–6.
Qu,F. et al. (2019) MicroRNA-497-5p down-regulation increases PD-L1 expression in clear cell renal cell carcinoma. J Drug Target, 27, 67–74.
Racle,J. et al. (2017) Simultaneous enumeration of cancer and immune cell types from bulk tumor gene expression data. eLife, 6, e26476.

Radovich,M. et al. (2018) The Integrated Genomic Landscape of Thymic Epithelial Tumors. Cancer Cell, 33, 244-258.e10.

Ritchie,M.E. et al. (2015) limma powers differential expression analyses for RNA-sequencing and microarray studies. Nucleic Acids Research, 43, e47–e47.

Robertson,A.G., Kim,J., et al. (2017) Comprehensive Molecular Characterization of Muscle-Invasive Bladder Cancer. Cell, 171, 540-556.e25.

Robertson,A.G., Shih,J., et al. (2017) Integrative Analysis Identifies Four Molecular and Clinical Subsets in Uveal Melanoma. Cancer Cell, 32, 204-220.e15.

Rooney,M.S. et al. (2015) Molecular and genetic properties of tumors associated with local immune cytolytic activity. Cell, 160, 48–61.

Ru,Y. et al. (2014) The multiMiR R package and database: integration of microRNA-target interactions along with their disease and drug associations. Nucleic Acids Res, 42, e133.

Rukov,J.L. et al. (2014) Pharmaco-miR: linking microRNAs and drug effects. Brief Bioinform, 15, 648–659.

Schmitz,R. et al. (2018) Genetics and Pathogenesis of Diffuse Large B-Cell Lymphoma. N Engl J Med, 378, 1396–1407.

Shen,H. et al. (2018) Integrated Molecular Characterization of Testicular Germ Cell Tumors. Cell Reports, 23, 3392–3406.

Smolle,M.A. et al. (2017) Noncoding RNAs and immune checkpoints-clinical implications as cancer therapeutics. FEBS J, 284, 1952–1966.

Spranger,S. et al. (2016) Density of immunogenic antigens does not explain the presence or absence of the T-cell-inflamed tumor microenvironment in melanoma. Proc. Natl. Acad. Sci. U.S.A., 113, E7759–E7768.

Srinivasan,G. et al. (2019) MiR223-3p promotes synthetic lethality in BRCA1-deficient cancers. Proc. Natl. Acad. Sci. U.S.A., 116, 17438–17443.

Sticht,C. et al. (2018) miRWalk: An online resource for prediction of microRNA binding sites. PLoS One, 13, e0206239.

Sturm,G. et al. (2019) Comprehensive evaluation of transcriptome-based cell-type quantification methods for immuno-oncology. Bioinformatics, 35, i436–i445.

Sturm,M. et al. (2010) TargetSpy: a supervised machine learning approach for microRNA target prediction. BMC Bioinformatics, 11, 292.

Su,R. et al. (2015) MiR-181 family: regulators of myeloid differentiation and acute myeloid leukemia as well as potential therapeutic targets. Oncogene, 34, 3226–3239.

Tibshirani,R. (1996) Regression shrinkage and selection via the lasso. J. R. Statist. Soc. B, 58, 267–288.

Tokar,T. et al. (2018) mirDIP 4.1-integrative database of human microRNA target predictions. Nucleic Acids Res, 46, D360–D370.

Tsang,J.S. et al. (2010) Genome-wide dissection of microRNA functions and cotargeting networks using gene set signatures. Mol Cell, 38, 140–153.

Vapnik,V. and Chervonenkis,A. (1974) Pattern Recognition. Theory, Statistical Learning Problems Nauka, Moskva.

Vasaikar,S. et al. (2019) Proteogenomic Analysis of Human Colon Cancer Reveals New Therapeutic Opportunities. Cell, 177, 1035-1049.e19.

Vejnar,C.E. and Zdobnov,E.M. (2012) MiRmap: comprehensive prediction of microRNA target repression strength. Nucleic Acids Res, 40, 11673–11683.

Wang,D. et al. (2014) OncomiRDB: a database for the experimentally verified oncogenic and tumor-suppressive microRNAs. Bioinformatics, 30, 2237–2238.

Wang,X. et al. (2015) Tumor suppressor miR-34a targets PD-L1 and functions as a potential immunotherapeutic target in acute myeloid leukemia. Cell Signal, 27, 443–452.

Xiao,F. et al. (2009) miRecords: an integrated resource for microRNA-target interactions. Nucleic Acids Res, 37, D105-110.

Xing,Y. et al. (2021) Tumor Immune Microenvironment and Its Related miRNAs in Tumor Progression. Front Immunol, 12, 624725.

Xu,J. et al. (2021) Immune-Related Nine-MicroRNA Signature for Predicting the Prognosis of Gastric Cancer. Front Genet, 12, 690598.

Yang,Q. et al. (2018) Regulation of cancer immune escape: The roles of miRNAs in immune checkpoint proteins. Cancer Lett, 431, 73–84.

Yi,M. et al. (2020) The role of cancer-derived microRNAs in cancer immune escape. J Hematol Oncol, 13, 25.

Yoav,F. and Schapire,R. (1997) A decision-theoretic generalization of on-line learning and an application to boosting. J. Comput. Syst. Sci., 55, 119–139.

Yue,D. et al. (2012) A Bayesian decision fusion approach for microRNA target prediction. BMC Genomics, 13 Suppl 8, S13.

Zheng,S. et al. (2016) Comprehensive Pan-Genomic Characterization of Adrenocortical Carcinoma. Cancer Cell, 29, 723–736.

Zou,H. and Hastie,T. (2005) Regularization and variable selection via the elastic net. J. R. Statist. Soc. B, 67, 301–320.

Zou,X. et al. (2021) Double Insurance for OC: miRNA-Mediated Platinum Resistance and Immune Escape. Front Immunol, 12, 641937.
